# Relationship between Impulsivity, Snack Consumption and Children's Weight

**DOI:** 10.1371/journal.pone.0088851

**Published:** 2014-02-19

**Authors:** Eline W. M. Scholten, Carola T. M. Schrijvers, Chantal Nederkoorn, Stef P. J. Kremers, Gerda Rodenburg

**Affiliations:** 1 IVO Addiction Research Institute, Rotterdam, The Netherlands; 2 Erasmus Medical Center, Rotterdam, The Netherlands; 3 Department of Experimental Psychology, Maastricht University, Maastricht, The Netherlands; 4 Department of Health Promotion, Maastricht University, Maastricht, The Netherlands; University of Missouri, United States of America

## Abstract

**Background:**

Childhood overweight is a public health problem associated with psychosocial and physical problems. Personality traits, such as impulsivity, may contribute to the development of overweight.

**Objective:**

This study examines 1) the association between general impulsivity traits (reward sensitivity and disinhibition) and children's weight, 2) the association between impulsivity traits and unhealthy snack consumption, and 3) the potential mediating role of unhealthy snack consumption in the relationship between impulsivity traits and children's weight.

**Methods:**

Included were 1,377 parent-child dyads participating in the IVO Nutrition and Physical Activity Child cohorT (INPACT). Children had a mean age of 10 years. Parents completed a questionnaire to measure children's unhealthy snack consumption. Children completed a door-opening task to assess reward sensitivity and completed a questionnaire to measure disinhibition. Children's height and weight were measured to calculate their BMI z-scores. Cross-sectional linear regression analyses were performed to test the associations.

**Results:**

Disinhibition was positively associated with unhealthy snack consumption but not with BMI z-scores. Reward sensitivity was not related to unhealthy snack consumption or to BMI z-scores.

**Conclusions:**

No evidence was found for a mediating effect of unhealthy snack consumption in the relation between impulsivity traits and children's weight. However, disinhibition appears to have a negative influence on children's unhealthy snack consumption. Future research focusing on food-related impulsivity in addition to general impulsivity will provide additional insight into factors that influence children's unhealthy snack consumption and weight.

## Introduction

Overweight and obesity in childhood has doubled in the past three decades [1]–[3]. Worldwide, about 10% of the children are overweight and the prevalence is even higher in the USA and Europe [4]. In the Netherlands in 2009, 13–15% of the children were classified as overweight, and 2% as obese [5], [6]. Overweight and obesity in children is an important public health problem, associated with a range of psychosocial and physical problems on the short and long term [7], [8]. Given the nature and extent of the problems related to overweight in childhood, it is important to develop intervention programs aimed at reducing childhood overweight. Therefore, more insight is needed into the mechanisms contributing to the development of childhood overweight.

One possible mechanism is related to impulsivity and unhealthy snack consumption. Impulsivity (considered to be a multidimensional construct) is a personality trait of acting on the spur of the moment, making hurried decisions and showing unpredictable behaviour [8]. Reward sensitivity (preference for immediate small rewards over delayed larger rewards) and disinhibition (low self-control) are aspects of impulsive behavior which are expected to be related to children's weight and eating habits [9]–[11]. It is known that both these impulsivity traits are associated with childhood weight: overweight children are more sensitive to reward and less effective in inhibiting responses than non-overweight peers [10], [12]–[15]. In addition, children with a high score on impulsivity exhibit more uncontrolled eating than children who score low on impulsivity [16], [17]. Finally, there is evidence that overeating mediates the relationship between impulsivity and children's weight [14]. Uncontrolled eating and overeating often involve energy-dense unhealthy food intake, including snacking [18], [19]. This suggests that unhealthy snack consumption can mediate the relationship between impulsivity and childhood weight.

Unhealthy snack consumption involves eating energy-dense food of little nutritional value between regular meals [19]. In general, eating unhealthy food is related to higher energy intake and overweight [17], [20]–[22]. However, cross-sectional and longitudinal studies provide conflicting results regarding whether children's unhealthy snack consumption contributes to childhood overweight [7]. Most studies investigating the association between children's unhealthy snack consumption and children's weight found no association [7], [19],[23]–[25]. This may be explained by measurement issues (e.g. the way in which unhealthy snack consumption is operationalized or the use of self-reports), but the relationship between impulsivity traits, unhealthy snack consumption and children's weight may also be dissimilar for overweight and non-overweight children. For example, for impulsive overweight children it may be more difficult to resist the temptation of snacks (e.g. potato crisps and candy) than for impulsive non-overweight children, resulting in more unhealthy snack consumption [9], [12], [14]. A Dutch longitudinal study showed that participants with the combination of a strong preference for unhealthy snacks and high disinhibition gained more weight than their peers [26]. These findings indicate that disinhibition makes people vulnerable to certain behaviour, whereas the manifestation is determined by domain-specific preferences (e.g. food preferences). It is also reported that food has a stronger rewarding value for overweight children than for non-overweight children [27], [28]. Therefore, the relationship between impulsivity and childhood weight, and the potential mediating effect of unhealthy snack consumption, may be stronger in overweight children than in their non-overweight peers.

**Figure 1:**
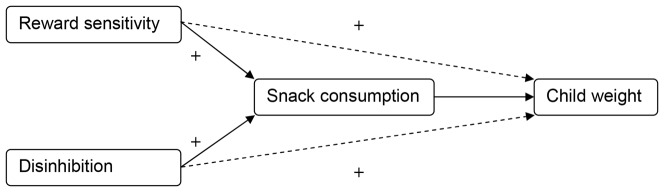
Conceptual research model.

The aim of the present cross-sectional observational study was to examine whether children's unhealthy snack consumption mediates the relationship of the two impulsivity traits, reward sensitivity and disinhibition, with children's weight (Figure 1). It was hypothesized that both impulsivity traits were positively related to children's weight (hypothesis 1) and unhealthy snack consumption (hypothesis 2). In addition, it was hypothesized that unhealthy snack consumption mediated the relationship of reward sensitivity and disinhibition with children's weight (hypothesis 3), meaning that (part of) the association between the impulsivity traits and children's weight is explained by children's unhealthy snack consumption. Evidence for a mediating effect of unhealthy snack consumption can contribute to the development of interventions aimed at reducing childhood overweight. The relatively stable impulsivity traits can help define at-risk children and an intervention can be applied to unhealthy snack consumption to control their weight. In the present study, the associations were examined in a sample of children aged 8–12 years, as well as in subsamples of non-overweight and overweight children.

## Methods

### Design, procedure and sample

The present study was conducted as part of the INPACT study. INPACT (IVO Nutrition and Physical Activity Child cohorT), approved by the Ethical Committee of the Erasmus Medical Centre, is a longitudinal study on dietary behaviour and physical activity of primary school children aged 8–12 years from the south of the Netherlands (Eindhoven and surroundings). In 2008, the primary schools were recruited in collaboration with the Municipal Health Authority for Eindhoven and surrounding area (GGD Brabant-Zuidoost). All 265 primary schools within this region were approached. A total of 91 (34.4%) schools agreed to participate in the cohort study. The response rate from schools in rural and urban areas was similar. In 2008, primary caregivers of third-grade students (aged about 8 years) were invited to participate in the study, together with their child. Of the 2948 invited parent-child dyads, 1840 (62.4%) gave written informed consent to participate in the four consecutive annual measurements starting in the autumn of 2008.

The present study was based on the third wave of data collection in which 1627 parent-child dyads participated. Parent-child dyads with no or invalid data on demographics (children's gender, age, ethnicity and socioeconomic status; SES) reward sensitivity, disinhibition, unhealthy snack consumption and BMI z-scores were excluded, resulting in 1377 included parent-child dyads. Attrition analysis showed that from baseline to 2010, parents from non-native Dutch children dropped-out more often than parents of native Dutch children. There was no selective dropout regarding children's gender, age and SES.

### Measurements

#### Reward sensitivity

To assess reward sensitivity, the children accomplished a door-opening-task, which is a computer task developed by Daugherty and Quay, in which children could open a virtual door by pressing a bar [29]. Behind the virtual door a happy or sad face appeared. A sad face meant losing one point and a happy face meant winning one point. Aim of the task was to earn as many points as possible and then stop the game. The chance of encountering a happy face diminished during the course of the task. The more doors the child opened, the fewer happy faces he/she encountered and the more points he/she lost. The children could stop the task whenever they wanted, and after completing the task they were praised for the number of points they earned. The more doors a child opened, despite all the losses, the more sensitive to reward he/she is. The scores ranged from 0–90 opened doors. This scale was used because previous studies showed that children with impulsive disorders score significantly higher on this task than children without impulsive disorders [12], [29]–[31]. This suggests that the door-opening-task is a valid and reliable scale to measure the impulsivity trait of reward sensitivity in children.

#### Disinhibition

The children completed a questionnaire to measure disinhibition. The questionnaire used to measure disinhibition contained 13 items of the Impulsivity Scale from the child reported version of the Temperament in Middle Childhood Questionnaire (TMCQ) [32]. This scale, translated in many languages, is a frequently used instrument to measure impulsivity in middle childhood, and research demonstrates that the child self-report scale reached high levels of internal consistency [33]. This suggests that children are a reliable source to report on their own impulsivity. We used the Dutch translated version of the scale, which is considered good in terms of internal consistency and factorial structure [34]. Example scale items are: “I say the first thing that comes into my head,” “I do things without thinking about them first,” “I grab what I want.” Per item, the children selected one of the following response categories: ‘never’ (1), ‘almost never’ (2), ‘sometimes’ (3), ‘usually’ (4), or ‘(almost) always’ (5). The item scores were reversed, so a low score on the items corresponded to a low degree of disinhibition. The 13 items were combined into one variable by averaging the item scores. The variable ranged from 1 (low) to 5 (high). The Cronbach's alpha (α = 0.73) and the mean item-total correlation (0.36) suggest a good consistency of the items. The variable is called disinhibition to make a clear distinction between the two impulsivity traits reward sensitivity and disinhibition. Where reward sensitivity is focused on the preference for immediate rewards, disinhibition is related to low self-control.

#### Unhealthy snack consumption

Unhealthy snack consumption of children was measured using a questionnaire based on the validated Food Frequency Questionnaire [35], [36] designed to accurately assess energy intake of Dutch children aged 2–12 [37], [38]. A validation study showed a correlation coefficient between the original questionnaire and the doubly labelled water method of 0.62, which implies that the questionnaire is a valid instrument to estimate energy intake in Dutch children [37]. In the present study, unhealthy snack consumption was assessed by the primary caregiver who reported how many days in a normal week his or her child eats 1) salty snacks (e.g. potato crisps or peanuts), 2) sweet snacks (e.g. candy or chocolate) and/or 3) large cakes or biscuits between the main meals. The response categories ranged from ‘never or less than 1 day a week’ to ‘7 days a week’. The primary caregivers also reported the number of units their children consume on such a day. The answer categories of this question ranged from 0–10 units a day. Children's unhealthy snack consumption was calculated by multiplying the frequency of the consumption of different types of snacks and the number of units the children consume on such a day. These scores were added to form a single score. The scores ranged from 0–40.

#### BMI z-score

According to standard procedures, the weight and height of children in light clothing and without shoes was measured by qualified research assistants at school. The weight and height of the children were used to calculate their BMI (weight/height^2^). BMI scores were standardized into z-scores [39] based on age and gender-specific values from the 1997 National Growth Study in the Netherlands [40]. These z-scores indicate by how many standard deviations (SD) a child's BMI differs from the median BMI of the reference population of his/her age. International cut-off scores were used to define subsamples of overweight and non-overweight children [39].

#### Confounders

Confounders of the association between the impulsivity traits and children's weight included children's gender, age, ethnicity and SES [41]–[43]. All potential confounders were parent-reported. Children's age was calculated in years by subtracting the children's date of birth from the date of completion of the questionnaire. Children's ethnicity was assessed based on the country of origin of both parents. According to standards procedures of Statistics Netherlands [44] a child was classified as ‘native Dutch’ (when both parents were born in the Netherlands) or as ‘non-native Dutch’ (when at least one of both parents was not born in the Netherlands). SES was based on the highest completed educational level of the primary caregiver. According to international classification systems, parental education level was defined as low (primary school and lower vocational/lower general secondary school), intermediate (intermediate vocational education, higher general secondary education and university preparatory) or high educational level (higher vocational education and university).

### Strategy for analyses

Descriptive analyses were performed to describe the distribution of children's age, gender, ethnicity, SES, reward sensitivity, disinhibition, unhealthy snack consumption and BMI z-scores. In addition, correlations between these variables were computed to explore the relationships.

The four-step approach of Baron and Kenny [45] was used to examine whether unhealthy snack consumption mediates the relationship of the impulsivity traits reward sensitivity and disinhibition, with children's weight. According to this approach, the predictor variables (i.e. reward sensitivity and disinhibition) and the outcome variable (i.e. BMI z-score) have to be associated (step 1); the predictor variables have to be associated with the potential mediator (i.e. unhealthy snack consumption) (step 2); and the potential mediator has to be associated with the outcome variable (i.e. BMI z-score) (step 3). The criteria for mediation are met when the associations in steps 1–3 are all statistically significant. Subsequently, step 4 assesses whether there is total or partial mediation. Therefore the association between the potential mediator and the outcome variable will be assessed when controlled for the predictor variable. To test if the criteria for mediation were met, several linear regression models were tested to assess the associations between:

*Step 1.* Both impulsivity traits (reward sensitivity and disinhibition impulsivity) and BMI z-scores (hypothesis 1).
*Step 2.* Both impulsivity traits and unhealthy snack consumption (hypothesis 2).
*Step 3.* Unhealthy snack consumption and BMI z-scores.
Step 4 was performed only when the criteria for mediation were met. The associations were tested in the total sample and all statistical analyses were repeated in a subsample of non-overweight children and in a subsample of overweight children. The analyses were conducted using IBM SPSS Statistics version 21.

## Results

### Descriptives

Table 1 presents the descriptive statistics. Most of the participating children (78.1%) were 10 years old (range 9–12, mean  = 10.2, SD = 0.5 years). Boys (50.8%) and girls (49.2%) were represented in almost equal numbers. Of all the children, 86.6% had a native Dutch ethnicity. Of the primary caregivers, 21.1% had finished education at the lowest level, 45.5% at intermediate level, and 33.4% at the highest level. The children opened on average 54.9 doors (range 2.0–90.0, SD = 23.3) and the mean disinhibition score was 2.5 (range 1.1–4.6, SD = 0.5). On average, children had a normal weight (range BMI z-scores −3.9–3.3 mean  = 0.0, SD = 1.0) and they consumed about 10 unhealthy snacks a week (range 0.0–37.0, mean  = 10.0, SD = 6.2).

**Table 1:** Descriptive statistics.

		Total sample (*n* = 1377)		Non-overweight children (*n* = 1199)		Overweight children (*n* = 178)	
		*n*	Proportion/Mean (SD)	*n*	Proportion/Mean (SD)	*n*	Proportion/Mean (SD)
*Demographics*							
Children's age							
	9–10 years	1127	81.8	993	82.8	134	75.3^a^
	11–12 years	250	18.2	206	17.2	44	24.7
Children's gender							
	Boy	699	50.8	624	52.0	75	42.1^b^
	Girl	678	49.2	575	48.0	103	57.9
Children's ethnicity							
	Native Dutch	1192	86.6	1056	88.1	136	76.4^c^
	Non-native Dutch	185	13.4	143	11.9	42	23.6
SES							
	Low	290	21.1	241	20.1	49	27.5^d^
	Middle	627	45.5	547	45.6	80	45.0
	High	460	33.4	411	34.3	49	27.5
*Study variables*							
Reward sensitivity			54.9 (23.3)		54.9 (23.4)		55.52 (22.5)
Disinhibition			2.5 (0.5)		2.5 (0.5)		2.47 (0.5)
Snack consumption			10.0 (6.2)		10.2 (6.1)		8.67 (6.3)
BMI z-scores			0.0 (1.0)		−0.2 (0.8)		1.73 (0.4)^e^

a Age_overweight_ > Age_non-overweight_, χ^2^ (1) = 5.9, *p*<0.05.

b Boy_overweight_ < Boy_non-overweight_, χ^2^ (1) = 6.1, *p*<0.05.

c Native Dutch_overweight_ < Native Dutch_non-overweight_, χ^2^ (1) = 18.2, *p*<0.01.

d SES is significantly different between non-overweight and overweight children, χ^2^ (2) = 6.2, *p*<0.05. It is not tested which specific SES groups differ between non-overweight and overweight children.

e BMI z-scores_overweight_ > BMI z-scores_non-overweight_, *t* (159.9) = −36.0, *p*<0.01.

Of the 1377 children, 178 (12.9%) were classified as overweight [39]. Children in the overweight subsample were relatively often of higher age, female, non-native Dutch and from low SES families, than children in the non-overweight subsample.

Table 2 provides correlations for the total sample of the demographic variables, reward sensitivity, disinhibition, unhealthy snack consumption and BMI z-scores. High reward sensitive children more often came from lower SES families than low reward sensitive children. Children with higher disinhibition scores were older, were more often male, and consumed more unhealthy snacks than children with lower disinhibition scores. Children from low SES families and children with low BMI z-scores consumed more unhealthy snacks than children from high SES families and children with higher BMI z-scores. Non-native children and children from low SES families had higher BMI z-scores than their peers. In the subsample of non-overweight children, the correlations of demographic variables and the main study variables were similar to the correlations in the total sample, except that the relation between children's age and BMI z-scores was weaker in the non-overweight subsample. Secondly, in the non-overweight subsample, boys had higher BMI z-scores than girls. Table 3 shows that in the subsample of overweight children, there were no significant associations between reward sensitivity and disinhibition on the one hand, and any of the other variables on the other. Older children and boys consumed more unhealthy snacks and had higher BMI z-scores than younger children and girls. Finally, children from high SES families had significantly lower BMI z-scores than children from lower SES families.

**Table 2:** Correlations between demographic variables and the main study variables in the total sample (*n* = 1377).

	1.	2.^c^	3.^c^	4.^c^	5.	6.	7.	8.
*Demographic variables*								
1. Children's age	-							
2. Children's gender^a^^,^ c	−0.03	-						
3. Children's ethnicity^b^^,^ c	0.09**	0.01	-					
4. Socioeconomic status^c^	−0.15***	−0.02	−0.03					
*Study variables*								
5. Reward sensitivity	0.01	0.02	0.05^∼^	−0.06*	-			
6. Disinhibition	0.06*	−0.17***	−0.02	−0.03	−0.02	-		
7. Snack consumption	0.02	−0.03	−0.01	−0.10***	0.02	0.06*	-	
8. BMI z-scores	0.05	−0.03	0.12***	−0.05*	0.02	0.04	−0.09**	-

*p<0.05;

**p<0.01;

***p<0.001.

a Boys are the reference category.

b Native Dutch children are the reference category.

c Spearman's rho correlation.

**Table 3:** Correlations between demographic variables and the main study variables in the subsample of overweight children (*n* = 178).

	1.	2.^c^	3.^c^	4.^c^	5.	6.	7.	8.
*Demographic variables*								
1. Children's age	-							
2. Children's gender^a^^,^ c	−0.19**	-						
3. Children's ethnicity^b^^,^ c	0.12	−0.12	-					
4. Socioeconomic status^c^	−0.17*	−0.03	0.02	-				
*Study variables*								
5. Reward sensitivity	0.00	−0.07	0.00	−0.07	-			
6. Disinhibition	0.03	−0.05	0.09	−0.02	−0.02	-		
7. Snack consumption	0.17*	−0.16*	0.06	−0.09	0.04	0.14	-	
8. BMI z-scores	0.27***	−0.37***	0.08	−0.25**	0.03	−0.02	0.09	-

*p<0.05;

**p<0.01;

***p<0.001.

a Boys are the reference category.

b Native Dutch children are the reference category.

c Spearman's rho correlation.

### Impulsivity traits and children's BMI

Table 4 presents results of the linear regression analyses for the total sample, for the subsample of non-overweight children, and for the subsample of overweight children. All analyses were adjusted for demographic variables. No statistically significant associations were found between the impulsivity traits reward sensitivity and disinhibition, and children's BMI z-scores.

**Table 4:** Linear regression analyses adjusted for socio-demographic variables^a^.

*Step 1.* The association between impulsivity traits and children's BMI z-scores			
	Total sample (*n* = 1377)	Subsample of non-overweight children (*n* = 1199)	Subsample of overweight children (*n* = 178)
	β	β	β
Reward sensitivity	0.01	0.02	−0.01
Disinhibition	0.04	0.03	−0.04
***Step 2.* Association between impulsivity traits and children's snack consumption**			
	**Total sample**	**Subsample of non-overweight children**	**Subsample of overweight children**
	**β**	**β**	**β**
Reward sensitivity	0.02	0.02	0.03
Disinhibition	0.05*	0.05	0.13
***Step 3.* Association between snack consumption and children's BMI z-scores**			
	**Total sample**	**Subsample of non-overweight children**	**Subsample of overweight children**
	**β**	**β**	**β**
Snack consumption	−0.10***	−0.06*	−0.02

β =  standardized regression coefficient.

^*^ *p*<0.05;

^**^ *p*<0.01;

^***^ *p*<0.001.

a All regression analyses adjusted for children's age, gender, ethnicity (native Dutch vs. non-native Dutch) and socioeconomic status.

### Impulsivity traits and children's unhealthy snack consumption

In the total sample, in contrast to reward sensitivity, disinhibition was significantly associated with unhealthy snack consumption. High scores on disinhibition were associated with more unhealthy snack consumption. In the subsamples of non-overweight and overweight children, disinhibition was not significantly related to unhealthy snack consumption.

### Unhealthy Snack consumption as a mediator of the association between impulsivity and weight

Regression analyses showed that more unhealthy snacking was associated with lower BMI z-scores in the total sample as well as in the subsample of non-overweight children. In the subsample of overweight children, the association of unhealthy snack consumption with BMI z-scores was not significant.

Due to the lack of statistically significant associations in steps 1–3 in the total sample, as well as in the subsamples, the mediation criteria were not met. Unhealthy snack consumption did not mediate the relation between the impulsivity traits and children's BMI z-scores.

## Discussion

The main aim of the present study was to examine whether children's unhealthy snack consumption mediated the relationship of the impulsivity traits and disinhibition with children's weight in the total sample, as well as in subsamples of non-overweight and overweight children. Contrary to our hypothesis, no mediating effect of unhealthy snack consumption was found. In the total sample, children who scored high on disinhibition consumed more unhealthy snacks than children who scored low on this impulsivity trait. In addition, children who consumed more unhealthy snacks were lower in weight than children who consumed less unhealthy snacks.

Our results imply that there is no relationship of the impulsivity traits reward sensitivity and disinhibition with children's weight. This is in contrast with our hypotheses and some previous studies [8], [10], [12], [13], [46]. The discrepancy in results between earlier studies and our study might be explained by differences in research methods. With regard to the relation between reward sensitivity and children's weight, previous research based on group comparisons demonstrated that obese children in treatment were more sensitive to reward than normal weight peers [12]. In the current study, however, a linear relationship was tested. In addition, we made no distinction between overweight and obese children, whereas in the latter study only obese children in treatment were compared with normal weight peers [12]. Studies showing a significant association between disinhibition and children's weight used stop-signal computer tasks to measure disinhibition [12], [13]. In the present study, the Impulsivity Scale from the child-reported version of the TMCQ was used, which is a valid scale to measure children's disinhibition [34]. In line with our results, an earlier study based on a go-no-go computer task to measure disinhibition also reported no statistically significant linear relationship [17]. Secondly, a linear relationship between impulsivity traits and children's weight may only exist in children who score above specific impulsivity cut-off scores. However, no international cut-off scores are currently available to assess impulsive children.

The hypothesis that impulsivity is positively related to unhealthy snack consumption is partly confirmed by the finding that impulsivity was positively associated with unhealthy snack consumption; this is in line with other studies [16], [17]. In our subsamples of non-overweight and overweight children this association was not significant. The standardized regression coefficient in the subsample of overweight children (β = 0.13) was higher than that in the total sample (β = 0.05) and in the non-overweight subsample (β = 0.05). This could indicate that the absence of a statistically significant relationship between disinhibition and unhealthy snack consumption in the overweight subsample may be due to loss of power in the subsample. Although statistically non-significant, the larger regression coefficient in the subsample of overweight children is in line with the notion that impulsive overweight children consume more unhealthy snacks than impulsive children without overweight [14].

In the current study, general impulsivity was assessed rather than food-specific impulsivity. It is reported that overweight children were (in particular) more impulsive than non-overweight children in response to food related stimuli [13] and that food has a stronger rewarding value for overweight children [27], [28]. The relationship between disinhibition and unhealthy snack consumption might have been stronger had we focused on food-specific impulsivity. In addition, this may also explain why no relationship was found between reward sensitivity and unhealthy snack consumption. A second explanation for this statistically non-significant relationship relates to our specific focus on children's unhealthy snack consumption (frequency and units) between regular meals, instead of studying a snacking or overeating eating pattern in general. Thirdly, instrumental feeding (whereby parents use food as a way to reward) is reported to be significantly related to children's unhealthy snack consumption [47]. The association between reward sensitivity and unhealthy snack consumption might be moderated by this parental feeding style and an association might only exist when parents reward their children with unhealthy snacks.

Contrary to our expectations, in the total sample and in the subsample of non-overweight children, children who consumed more unhealthy snacks had lower weight than children who consumed less unhealthy snacks. These findings are in line with an earlier study showing that the prevalence of children's overweight decreased with increased snacking frequency and with increased percentage of energy from snacks [46]. There are several possible explanations for this negative association. Firstly higher weight children may actually consume less unhealthy snacks than their lower weight peers; due to their weight, their unhealthy snack consumption might be more restricted by parental influence than that of their peers [48], [49]. Secondly, not just unhealthy snack consumption, but rather the overall eating pattern and energy intake seem important in children's weight [50]. A third explanation may be that lower weight children compensate their unhealthy snack consumption more than higher weight children, both in their energy intake during the remainder of the day and in their physical activity [51]–[53]. Fourthly, high weight can partly be explained by biological predisposition [53]–[55], body density [56] and metabolic processes [54], [55]. It is possible that, due to genetic factors, individual differences in body density and individual differences in metabolic processes, unhealthy snack consumption does not have equal weight consequences for every child. Remarkably, in the subsample of overweight children, the association between unhealthy snack consumption and children's weight was not statistically significant, implying that the association between unhealthy snack consumption and children's weight in this subsample differs from that in the total sample and in the subsample of non-overweight children. This is in line with our hypothesis that the relation between unhealthy snack consumption and children's weight may be different for overweight and non-overweight children. To improve our understanding of the relationship between unhealthy snack consumption and child weight, conditions which may confound the relationship, such as total energy intake, physical activity levels and parental influence, should be taken into account in future studies.

### Strengths and limitations

The present study examined a possible mechanism which might contribute to the development of children's overweight. The associations were tested in a large sample of 1377 children, as well as in subsamples of non-overweight (*n* = 1199) and overweight children (*n* = 178). Our study is unique in that a large number of children accomplished the computer task that we used to measure reward sensitivity.

The current study also has some limitations. First, the cross-sectional design means that we cannot make statements about causality or bidirectional relationships. However, a bidirectional relationship between impulsivity, and unhealthy snack consumption and children's weight is not expected, because impulsivity can be seen as a relatively stable personality trait [57]. However, the relationship between unhealthy snack consumption and children's weight could be bidirectional. Higher unhealthy snack consumption may influence children's weight, but actual weight and weight perception may also influence children's unhealthy snack consumption [58]–[60]. Secondly, children's unhealthy snack consumption is measured based on the Food Frequency Questionnaires, filled out by the parents. It is possible that social desirability answering led to underestimation of total unhealthy snack consumption, especially in the subsample of overweight children [50], [61], [62]. This could distort the relationship of impulsivity traits with unhealthy snack consumption, and with children's weight. However, there is evidence that selective underreporting in overweight children does not occur when parents report their children's unhealthy snack consumption, which was the case in the present study [63]. It remains unclear to what extent parents are fully informed of the total unhealthy snack consumption of their children. However, this applies to parents of all the children and there is no indication that this led to selective underreporting. Thirdly, although analyses showed selective dropout on ethnicity, selective dropout is unlikely to have distorted our results because ethnicity was not a main predictor in our study.

### Conclusion

No indication was found for a mediating effect of unhealthy snack consumption in the relation of the impulsivity traits with children's weight. However, disinhibition may have a negative influence on children's unhealthy snack consumption. Reward sensitivity was not associated with unhealthy snack consumption. For future research it seems relevant to investigate food-related impulsivity in addition to general impulsivity in relation to unhealthy snack consumption and child weight. Also, unhealthy snack consumption was found to be negatively associated with children's weight in the total sample and in the subsample of non-overweight children, but not in the overweight subsample. Our results imply different associations between unhealthy snack consumption and children's weight in the different subgroups. To gain insight into the processes that might contribute to overweight, future research could investigate associations in different weight-based subgroups and focus on different components of children's eating patterns.
